# Prospects of POLD1 in Human Cancers: A Review

**DOI:** 10.3390/cancers15061905

**Published:** 2023-03-22

**Authors:** Michał Gola, Przemysław Stefaniak, Janusz Godlewski, Barbara Alicja Jereczek-Fossa, Anna Starzyńska

**Affiliations:** 1 Department of Human Histology and Embryology, Collegium Medicum, School of Medicine, University of Warmia and Mazury, 10-082 Olsztyn, Poland; 2 Department of Surgical Oncology, Hospital Ministry of Internal Affairs with Warmia and Mazury Oncology Centre, 10-228 Olsztyn, Poland; 3 Division of Radiation Oncology, European Institute of Oncology (IEO), Istituto di Ricovero e Cura a Carattere Scientifico (IRCCS), 20141 Milan, Italy; 4 Department of Oncology and Hemato-Oncology, University of Milan, 20122 Milan, Italy; 5 Department of Oral Surgery, Medical University of Gdańsk, 7 Dębinki Street, 80-211 Gdańsk, Poland

**Keywords:** DNA repair, polymerase delta, germline and somatic mutations, DNA replication

## Abstract

**Simple Summary:**

Polymerase delta 1 (POLD1), a catalytic and proofreading subunit of the human DNA polymerase delta (Polδ), plays a vital role in maintaining the stability of the genome. In recent years, *POLD1* germline and somatic mutations, as well as gene-expression patterns, have been extensively studied in cancers. Nevertheless, the complex regulatory mechanisms of *POLD1* expression and their clinical relevance are yet to be elucidated. This review aims to summarize the current research status regarding the role of POLD1 in neoplastic processes.

**Abstract:**

Cancer is the second leading cause of death globally, exceeded only by cardiovascular disease. Despite the introduction of several survival-prolonging treatment modalities, including targeted therapy and immunotherapy, the overall prognosis for the metastatic disease remains challenging. Therefore, the identification of new molecular biomarkers and therapeutic targets related to cancer diagnosis and prognosis is of paramount importance. DNA polymerase delta 1 (POLD1), a catalytic and proofreading subunit of the DNA polymerase δ complex, performs a crucial role in DNA replication and repair processes. Recently, germline and somatic mutations of the *POLD1* gene have been acknowledged in several malignancies. Moreover, diversified *POLD1* expression profiles have been reported in association with clinicopathological features in a variety of tumor types. With this review, we aim to summarize the current knowledge on the role of POLD1 in cancers. In addition, we discuss the future prospects and clinical applications of the assessment of *POLD1* mutation and expression patterns in tumors.

## 1. Introduction

Within the next twenty years the number of new cancer cases is expected to nearly double globally [1]. In addition, the observed increase in incidence will presumably be paralleled by a rise in cancer-related deaths [1]. The projected increase is expected to be driven by both aging and the growth of the human population worldwide [1]. Despite the rapid progress in cancer diagnosis and treatment, when it is diagnosed at a metastatic stage the prognosis remains poor [2]. Therefore, the identification of novel molecular diagnostic, prognostic, and predictive biomarkers may be highly warranted. 

The polymerase delta 1 (*POLD1*) gene encodes the major catalytic and proofreading subunit of the DNA polymerase δ (Polδ) holoenzyme [3]. It is crucial that *POLD1* expression is precisely regulated during the cell cycle [4]. With regard to the Polδ complex, it plays an essential role in DNA replication as it synthesizes the DNA at the lagging strand. Furthermore, the important error-correcting ability provided by the exonuclease activity of this enzyme contributes significantly to replication fidelity [5]. In addition, Polδ is involved in DNA repair upon exposure to mutagens [6]. 

Both germline and somatic alterations in the proofreading domain of *POLD1* have been implicated in various diseases, including cancers [7]. Moreover, *POLD1* mutation is thought to be strongly associated with genomic instability, mutator phenotype, and tumorigenesis [7,8,9].

Although *POLD1* germline mutation was primarily linked to colorectal and endometrial cancers, more recent data have also found a connection between this mutation and an increased risk of many other tumor types [7,10]. Notably, two clinical conditions regarding the cancer-predisposing nature of the *POLD1* mutation have been mentioned in the literature: the first, defined as polymerase proofreading-associated polyposis [11] and the second, the recently described POLE/POLD1-associated tumor syndrome, which have expanded the spectrum of possible tumors affecting people with inherited DNA polymerase-related mutations [12]. Nonetheless, somatic mutations of the *POLD1* gene are inadequately studied in cancer [7]. Thus, they warrant further investigation to be appropriately addressed clinically and practically.

As for immunohistochemical POLD1 protein expression levels in malignancies, their correlation with clinicopathological characteristics has lately been confirmed [13,14,15,16,17,18,19,20,21,22,23,24,25]. Notably, the downregulation of *POLD1* expression also occurs under physiological conditions, such as replicative senescence to mediate aging [26]. Advancing our understanding of the *POLD1* expression patterns in various cancer types in relation to meaningful clinical and pathological parameters may facilitate the translation of research knowledge into clinical practice.

Past articles have thoroughly reviewed the role of POLD1 in tumors in 2016 [7,9]. Therefore, our aim is to summarize and update the current understanding of the *POLD1* mutations and expression changes in human cancers. Moreover, we address possible therapeutic implications and future challenges in *POLD1*-mutated cancers.

## 2. *POLD1* Gene and a Polymerase Delta Holoenzyme in Health

The DNA polymerase delta 1 catalytic subunit (*POLD1*) gene is located on chromosome 19 at q13.3-q13.4 [27,28]. The human *POLD1* gene is composed of 27 exons and 26 introns. It encodes a large 125-kDa protein known as the POLD1/p125 subunit, a catalytic and proofreading subunit of the human DNA polymerase delta (Polδ). The fluctuations of the *POLD1* promoter activity imply that the transcriptional mRNA and protein levels are regulated during the cell cycle [4]. The *POLD1* gene promoter is activated by Sp1, Sp3 transcription factors, cell cycle-related E2F1, and CCCTC-binding factor (CTCF) [29,30]. On the other hand, the p53 protein, the “genome guardian”, and cyclin-dependent kinase inhibitor p21 are able to inhibit the Sp1-stimulated *POLD1* promoter activity, thus repressing *POLD1* gene expression [31,32].

Song et al. discovered that a cell cycle-dependent element (CDE)/cell cycle gene homology region (CHR) element—is an active functional element in the *POLD1* promoter, crucial for the cell cycle regulation of the *POLD1* gene [32]. They also showed that E2F1 and p21 regulate the *POLD1* promoter via binding to the CDE/CHR element [32]. Interestingly, p53-mediated inhibition of *POLD1* transcription seems to be indirect [33]. p53 does not directly bind to the CDE–CHR motifs. Instead, p53 triggers indirect p21-dependent *POLD1* downregulation via the p53–p21–DREAM–CDE/CHR pathway [33]. In addition, CTCF mainly binds to the *POLD1* promoter in site 3 and 4 regions [30]. It has been shown that these two regions play crucial roles in CTCF-mediated *POLD1* regulation [30]. Additionally, microRNA-155-mediated suppression of the forkhead transcription factor FOXO3a subsequently contributes to the decreased expression of *POLD1* at the mRNA and protein levels [34].

Moreover, *POLD1* expression has been causally linked to age-related cellular senescence and two POLD1-related mechanisms of aging have been proposed [26,30]. Gao et al. showed that the attenuation of the binding affinity of E2F1 for the *POLD1* promoter, via age-related decline in E2F1 expression and increased CpG island 3 methylation in the *POLD1* promoter region, causes a reduction in *POLD1* expression in replicative senescence [26]. Hou et al. proposed, however, that an age-related decline in CTCF levels affects the binding affinity of CTFC to the *POLD1* promoter, thus driving down *POLD1* expression [30]. It is, therefore, well established that down-regulation of *POLD1* is a characteristic age-related change occurring in humans [26,30,35].

Human Polδ holoenzyme has been believed to be a heterotetramer in unstressed, non-dividing cells composed of a catalytic subunit (POLD1) and three accessory subunits, POLD2 (p50), POLD3 (p66/p68), and POLD4 (p12) [36]. However, it has recently been suggested that the POLD4 subunit may occur as a dimer in the human Polδ complex [37]. Therefore, human Polδ potentially exists under a pentameric structure [37]. Through the catalytic POLD1 subunit, in association with both replication factor C (RFC) and proliferating cell nuclear antigen (PCNA), Polδ plays a pivotal role in discontinuous DNA synthesis at the lagging strand during genome replication. Moreover, its 3′–5′ exonuclease domain provides an error-correcting activity during DNA synthesis [38]. It has also been shown that Polδ is required for break-induced telomere synthesis [39].

A pivotal role for POLD1 in maintaining the enzymatic function of the Polδ complex suggests that adequate cellular *POLD1* expression levels should be sustained for the smooth functioning of genomic DNA replication [40]. Novel data suggest that POLD1 is protected from degradation while being interconnected with the three different subunits in the Polδ complex, whereas excessive non-associated POLD1 is degraded to intercept the formation of incomplete and inactive Polδ units [40]. Moreover, results obtained in an experimental study using a murine model revealed the overall low levels of POLD1-3 subunits in POLD3-deficient cells [41]. 

**Figure 1:**
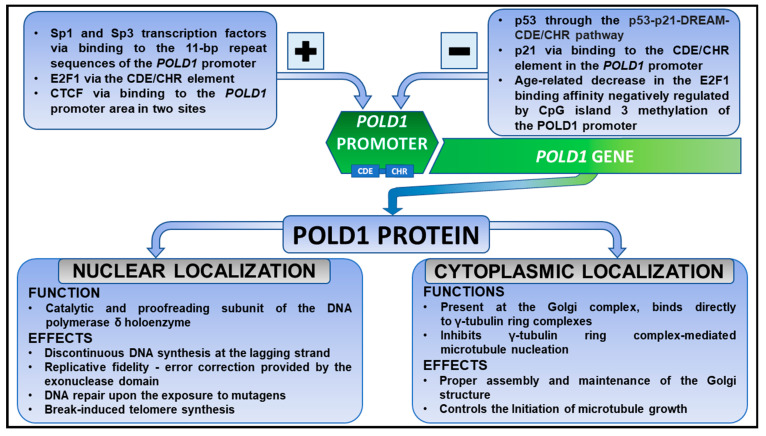
Regulation of *POLD1* gene expression within the cell and physiological roles of POLD1 depending on its cellular localization. The figure highlights positive and negative regulators of the *POLD1* promoter. Apart from well-known functions of POLD1 exhibited in the nucleus, recent studies have also noticed its role within the cytoplasm. bp, base pair; CDE/CHR, cycle-dependent element/cell cycle gene homology region; CTCF, CCCTC-binding factor; DREAM, dimerization partner, RB-like, E2F, and MuvB core complex.

Surprisingly, it has recently been shown that POLD1 plays a unique role in both the nucleus and the cytoplasm [42]. Subcellular fractionation has revealed that more than 60% of the POLD1 content occurs in the cytoplasm [42]. Regarding its cytoplasmic function, the POLD1 protein localized at the Golgi complex controls microtubule growth [42]. Shen et al. demonstrated that POLD1 undergoes bidirectional nucleocytoplasmic transport [43]. In addition, they also substantially unraveled the mechanisms underlying both the nuclear import and export of the POLD1 protein [43]. Figure 1 summarizes the mechanisms of *POLD1* gene regulation. Moreover, it outlines the physiological role of the POLD1 protein within the cytoplasm and nucleus.

Interestingly, during normal cell cycle progression, conversion of heterotetrameric Polδ (Polδ4) to the heterotrimer (Polδ3) takes place via POLD4/p12 subunit degradation [40,41]. It has been shown that POLD4 undergoes proteasomal degradation initiated by its polyubiquitination, in synchrony with the S phase [44,45]. Contrarily, no alteration in POLD1, POLD2, and POLD3 levels with the progression of the cell cycle was observed in Zhang et al.’s studies [45]. Although both Polδ4 and Polδ3 are active polymerases, Polδ3 seems to be the predominating form of human Polδ during the S phase of the cell cycle [6,45]. Moreover, a direct comparison of Polδ3 with Polδ4 has shown that the heterotrimeric complex displays a higher ratio of exonuclease to polymerase activity in vitro than four-subunit Polδ4 [6]. In addition, POLD4 degradation in response to DNA damage after the application of ultraviolet, methyl methanesulfonate, hydroxyurea, and aphidicolin, causes the concomitant conversion of Polδ4 to a heterotrimeric form [46]. Therefore, exposure to the agents causing DNA damage following Polδ4 to Polδ3 conversion suppresses DNA synthesis, while at the same time favoring proofreading activity under conditions of DNA replication stress. 

## 3. *POLD1* Gene and a Polymerase Delta Holoenzyme in Non-Oncogenic Processes

Numerous studies have documented the physiological role of both Polδ holoenzyme and POLD1 protein. As they are involved in multiple biological processes, including DNA replication and DNA repair, a growing body of studies in recent years has highlighted the role of germline and somatic mutations in *POLD1*–*POLD4* genes in the genesis of human pathologies. Mandibular hypoplasia, deafness, progeroid features, and progressive lipodystrophy (MDPL) syndrome is a rare autosomal dominant disorder caused by heterozygous de novo mutations in the *POLD1* gene [47,48]. Notably, exome sequencing revealed that mutations in different POLD1 domains may lead to a major phenotypic variability in MDPL syndrome [48]. Recent data have also revealed that *POLD1* mutations causing reduced Polδ polymerase activity with a maintained exonuclease activity can lead to a non-syndromic sensorineural hearing loss in an autosomal recessive manner [49]. In addition, several cases of autosomal recessive *POLD1*-linked combined immunodeficiency have been reported [50,51,52]. Additionally, Nichols-Vinueza et al. described a potential role for POLD1 in B-cell maturation [52].

## 4. POLD1 in Cancers

Knowing that Polδ is crucial in genome maintenance and is involved in the DNA proofreading processes, it is not surprising that mutations in the *POLD1* gene have been linked to genomic instability, mutator phenotype, and carcinogenesis [7,8,9]. In this paragraph, we aim to discuss the influence of both germline and somatic *POLD1* mutations on different malignancies. Moreover, the changes in the *POLD1* expression level in relation to clinicopathological parameters will be addressed.

### 4.1. Colorectal Cancer

Colorectal cancer (CRC) is the third most frequently diagnosed cancer worldwide but is second in terms of mortality [1]. Almost a third of all CRC cases have a familial component, however, only about 5% of these develop as a consequence of well-defined inherited syndromes, whereas the majority of them do not possess a known genetic background [53,54]. To date, colorectal cancer (CRC) seems to be the most extensively studied malignancy concerning *POLD1* mutations. 

Among patients with a family history of CRC and multiple or large colorectal polyps, a minor fraction of carriers of *POLD1* heterozygous germline mutations can be found [55,56]. Regarding somatic pathogenic variants affecting *POLD1* in CRC, they are thought to be extremely rare findings [57]. Unfortunately, the overall incidence of *POLD1* mutation in CRC patients remains unknown. Since the first description in 2013 of a correlation between the *POLD1* mutation and dominantly inherited intestinal adenomas and carcinomas [55], two terms regarding this phenomenon have been coined. Originally, this syndrome was described as “polymerase proofreading-associated polyposis” [11], however, the term “POLE/POLD1-associated tumor syndrome” was recently proposed instead [12], as among some patients with a germline mutation in DNA polymerase subunits *POLE* and/or *POLD1*, expanded extracolonic tumor spectrum and absence of colonic polyposis have been noted. Interestingly, tumors with *POLE*/*POLD1* mutations were found to be hypermutated, chromosomally unstable, and, surprisingly, microsatellite stable [55]. Moreover, recent data suggested the occurrence of an abundance of tumor-infiltrating lymphocytes (TILs) in *POLE*/*POLD1*-mutant CRCs [58]. In general, lymphocytes located in the tumor tissue region have emerged as the activators of a patient’s pre-existing intratumoral immunity after exposure to ICI treatment [58].

Unfortunately, data regarding *POLE*/*POLD1* status to predict the efficacy of immunotherapy in CRC treatment are scarce, thus conducting research in this field would be reasonable.

Recently, Siraj et al. investigated the correlation of immunohistochemical POLD1 protein expression with clinicopathological features in Middle Eastern CRCs [13]. They observed that low *POLD1* expression, found in more than half of CRC cases, was correlated with larger tumor size, adenocarcinoma histology, and stage III tumors [13]. Moreover, in our study focused on patients with CRC using IHC analysis, lower *POLD1* nuclear expression in CRC cells compared with normal epithelial colon cells was observed. *POLD1* expression levels in the tumor cells did not correlate with clinicopathological factors and the prognosis of CRC patients (data not published yet).

**Figure 2:**
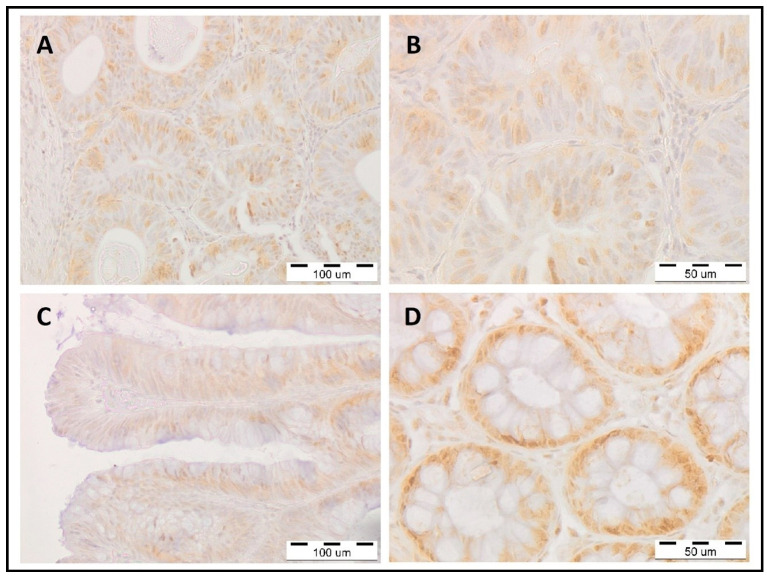
Immunohistochemical staining against POLD1 within tissues of the human large intestine. (**A**,**B**) Represent *POLD1* expression in the colon cancer tissue; (**C**) *POLD1* expression in villous adenomas; (**D**) *POLD1* expression in epithelial cells of normal mucosa of the large intestine, view of the transverse section of intestinal crypts. All sections were stained using immunohistochemical methods: primary antibody—rabbit anti-human antibody against POLD1 (HPA046524; Sigma-Aldrich, St. Louis, MO, USA), next—secondary antibody HRP-conjugated (ready-to-use dilution; ImmPRESS Universal reagent Anti-Mouse/Rabbit Ig, Vector Laboratories). Finally, the sections were stained in diaminobenzidine (DAB; Dako, Glostrup, Denmark) and counterstained with hematoxylin (Sigma-Aldrich, St. Louis, MO, USA). The magnification was (**A**,**C**) 200× and (**B**,**D**) 400×; particular bars are labeled in each photo.

In Figure 2, we present representative immunohistochemical staining patterns of POLD1 within healthy, benign polypoid, and malignant tissues.

### 4.2. Endometrial Cancer

With its incidence continuing to rise, endometrial cancer (EC) is the most common gynecological malignancy in developed countries [1,59]. Although the *POLD1* mutations in general are very scarce in EC [14,60], it is now well established that *POLD1* germline mutations predispose people to EC [11,14]. Presumably, the frequency of *POLD1* mutations in EC may be population dependent [60,61]. Among 47 South East Asian women with grade 3 (according to FIGO grades) endometrioid ECs, a presence of two pathogenic germline *POLD1* mutations was detected [61]. Whereas Church et al.’s data revealed only one germline *POLD1*-mutated endometrioid EC among 154 patients with different FIGO grades enrolled in a British study [60].

Moreover, EC was found to be the most common malignancy among female *POLD1* germline mutation carriers [62]. Palles et al.’s studies revealed that EC is the most frequent extraintestinal cancer in polymerase proofreading-associated polyposis syndrome [62]. Surprisingly, one recent study on Chinese genomic cancer population data suggests that EC may be the most common malignancy with *POLD1* mutations among all cancers [63].

Even though *POLD1*-mutated cancers are believed to be primarily microsatellite stable [55], some ECs and CRCs possess microsatellite instability (MSI) [63,64,65,66,67]. Notably, Haraldsdottir et al. have suggested that the deficient DNA mismatch repair system in endometrial and colorectal cancers resulting in MSI phenotype, may occur mainly in tumors with somatic rather than germline *POLD1*/*POLE* mutations [64]. In addition, one study that assessed an endometrial dedifferentiated carcinoma molecular profile suggested that *POLD1* and *MLH1* (a gene involved in DNA mismatch repair), as well as high microsatellite instability (MSI-H) and increased tumor mutation burden (TMB), may be peculiar to this rare distinct subtype of EC [68].

As it is widely known that patients with MSI-H tumors can benefit from immunotherapy [69], routine clinical genetic testing for both *POLD1*/*POLE* and MSI-related mutations may help identify patients with endometrial and colorectal cancers who could respond well to immunotherapy [70]. To date, only one study evaluated POLD1 protein immunohistochemical expression in EC and its association with the clinicopathological profile [14]. Low expression of *POLD1* was noted in almost 60% of Saudi EC cases and significantly correlated with grade 1 tumors [14].

Nevertheless, conducting research to establish both prognostic and predictive implications of *POLD1* mutation and/or POLD1 protein expression for EC patients is justified.

### 4.3. Renal Cancer

Clear cell renal cell carcinoma (ccRCC) is the most common histopathological subtype of renal cancer, accounting for up to 85% of all kidney cancers [71]. Current studies have highlighted the role of DNA damage repair pathways in ccRCC. Two research groups have recently analyzed DNA polymerase epsilon and delta and their possible prognostic significance for patients with ccRCC [15,72]. Our team was the first to report that, unexpectedly, POLD1 nuclear protein expression levels correlated with better prognosis in patients with ccRCC [15]. Moreover, we have also demonstrated that the level of POLD1 immunoexpression in tumor cells does not correlate with the demographic and clinicopathological characteristics of ccRCC patients [15]. This unanticipated data demonstrating the positive correlation of POLD1 nuclear immunoreactivity with longer overall survival among ccRCC patients, in contrast to several reports regarding other malignancies, may suggest that the potential role of POLD1 can be considered as cancer specific [14,17]. 

Eventually, subsequent molecular studies will be crucial to clarify the role of POLD1 in ccRCC tumorigenesis.

### 4.4. Liver Cancer

Hepatocellular carcinoma (HCC), accounting for 75–85% of primary liver cancers [1], is the second most lethal malignancy after pancreatic cancer, with a 5-year relative sur-vival of 18% [59]. The role of POLD1 in HCC was first presented in 2010 [16]. Sanefuji et al. demonstrated that *POLD1* overexpression induced by tp53 mutation correlates with a high degree of vascular invasion, poor cellular differentiation, and a poorer prognosis in HCC [16]. They have also suggested that POLD1 may contribute to cancer cell dedifferen-tiation in human HCC [16]. Furthermore, apoptosis of HCC cells was shown to be related to the inhibition of *POLD1* gene expression [73]. Similarly, apoptosis of triple-negative breast cancer (BC) cells was driven by the reduction in *POLD1* expression [74]. 

Recently, Tang et al. conducted a thorough analysis of the predictive and prognostic value of POLD1 in HCC [17]. It was shown that *POLD1* is significantly upregulated in HCC in comparison with adjacent healthy liver tissue [17]. Additionally, *POLD1* overexpression correlated with increased levels of alpha-fetoprotein, high TNM stage (III and IV), and predicted worse prognosis in HCC [17]. In addition, aberrantly high expression of *POLD1* in HCC tissue was demonstrated to be caused by DNA copy number gain, low *POLD1* methylation, and downregulation of small noncoding miR-139-3p [17]. Lastly, *POLD1* expression positively correlated with the immune infiltration levels of B cells, macrophages, dendritic cells, and CD4+ T cells in the HCC tumor microenvironment [17]. Moreover, an in vivo orthotopic liver injection model revealed that POLD1 knockdown in HCC cells diminished tumor incidence, size, and lung metastases in mice [75].

Taken together, an in-depth exploration of HCC tumorigenesis can aid in the development of novel clinical treatment modalities.

### 4.5. Breast Cancer

With an estimated 2.3 million new cases in 2020, breast cancer is the current leading cause of global cancer incidence, constituting 11.7% of all cancers diagnosed worldwide [1]. Patients harboring germline *POLD1* mutations are at higher risk of developing BC compared with the general population [76,77]. Somatic mutations, although insufficiently documented, have also been reported in BC [78,79]. Furthermore, even though BC cases are infrequently associated with MSI [80], this particular group of tumors may be *POLD1*-mutated as well [81].

Research conducted by Zhang et al. highlighted the mechanisms underlying changes in *POLD1* expression in BC [18]. They have shown that *POLD1* expression and the methylation level of the *POLD1* promoter are both increased in BC tissues and cell lines [18]. Moreover, *POLD1* expression and the methylation of the *POLD1* gene promoter were found to be inhibited by the p53 protein through the suppression of DNA methyltransferase 1 and Sp1 activities in BC [18]. In the following studies, they observed that sirtuin 1, a member of the sirtuin family, enhances proliferative, migratory, and invasive properties of BC cells in vitro by inhibiting the p53 protein expression and, therefore, promoting the *POLD1* upregulation [82].

To date, data regarding the expression patterns and clinical value of *POLD1* among patients with BC are scarce. However, novel studies conducted by Qin et al. revealed that increased *POLD1* expression is linked to poor prognosis in BC [19]. Elevated POLD1 levels were related to lymph node metastasis, histological grade, p53 status, and the ki-67 index [19]. Moreover, survival analysis showed that BC patients with high POLD1 levels had poorer disease-free survival in comparison with patients with low *POLD1* expression [19]. In addition, *POLD1* status likely affects the treatment response, especially in triple-negative breast cancer [83].

Thus, these data suggest that the POLD1 protein can be considered as a potential prognostic biomarker in BC [19].

### 4.6. Other Cancers

Although *POLD1* mutations in cancers seem to be understudied, they may serve as promising prognostic/predictive biomarkers in the future and, importantly, future targets for novel POLD1-oriented therapies. Nevertheless, apart from malignancies mentioned in the previous paragraphs, several different cancers have been reported to harbor *POLD1* mutation as well. Wang et al.’s studies that aimed to assess the clinical benefits of immune-checkpoint inhibitor (ICI) treatment have estimated that across almost 50,000 patients with different cancer types, *POLD1* mutation occurred in 1.37% of cases [10].

For instance, the overall frequency of *POLD1* mutations was estimated to be 2.5% among Chinese patients with lung cancer [84]. Furthermore, a recent analysis revealed that POLD1 is associated with the development of non-small cell lung cancer (NSCLC) from chronic obstructive pulmonary disease [20]. Interestingly, high expression levels of *POLD1* indicated poor prognosis in lung adenocarcinoma but not in squamous cell carcinoma [20]. Additionally, in both mesothelioma, a rare and deadly cancer, and esophageal squamous cell carcinoma, POLD1 has been found to play a significant role in resistance to platinum-based chemotherapy [85,86]. 

In papillary thyroid cancer, low *POLD1* expression implies poor clinicopathological characteristics [21]. Recently, POLD1 protein level was shown to be negatively correlated with the expression of cadherin-16 in this cancer [87].

In gastric adenocarcinoma, *POLD1* mutation occurred in 2.77% of 613 patients [88]. Zhu et al. have also shown that using immunotherapy in *POLE*/*POLD1*-mutated tumors may have potentially positive implications [88].

Comprehensive molecular profiling has identified the presence of *POLD1* mutations in brain tumors [62,89,90,91]. Additionally, *POLD1* expression in this heterogeneous group of neoplasms was only described in 1p19q co-deleted lower-grade gliomas [24]. Yet, the clinical significance of *POLD1* mutations and changes in the POLD1 protein level in central nervous system tumors is not fully understood. 

Studies concerning uveal melanoma, the most common primary intraocular malignancy in adults, have discussed the changes in *POLD1* expression patterns within patients with this cancer [22,23].

Much research has been conducted to investigate the possible clinical significance of POLD1 in gynecologic malignancies. Besides endometrial carcinoma, cervical and ovarian cancers were also assessed for the importance of *POLD1* mutations and expression changes [92,93]. Interestingly, the *POLD1* gene was more frequently altered among Chinese women with locally advanced cervical cancer than in Caucasian patients [92]. Furthermore, using cisplatin-resistant and cisplatin-sensitive ovarian cancer cell lines, Xing et al. demonstrated that *POLD1* was highly expressed in the platinum-sensitive group, whereas chemoresistant cell lines possessed a reduced expression of *POLD1* [93]. 

Intriguingly, in acute lymphoblastic leukemia (ALL), *POLD1* upregulation may promote the relapse of this disease [25]. Thus, its expression could be proposed as the potential diagnostic marker and therapeutic target for the treatment of relapsed ALL [25].

A summary of studies discussing the relationship of *POLD1* expression level with clinicopathological characteristics was provided in Table 1.

## 5. Conclusions and Future Directions

With cancer incidence rates continuing to rise globally, there is a strong need for the development of new biomarkers and treatment strategies [1]. The genetic mutations and expression profiles in *POLD1*-mutated cancers have recently gained remarkable attention. Nevertheless, the complex regulatory mechanisms of *POLD1* expression in cancers remain to be fully characterized.

Both *POLD1* germline and somatic mutations within the proofreading domain are considered rare in a variety of malignancies [9,10,63]. Additionally, the presence of pathogenic *POLD1* germline alterations has been linked with some hereditary tumors, such as colorectal and endometrial cancers [55]. 

Based on the primary role of Polδ in genome maintenance, mutations in the catalytic *POLD1* subunit lead to genomic instability, mutator phenotype, and malignant transformation [7,9]. Missense mutations are believed to be the most common mutations in *POLD1*-associated tumors, and no mutation hotspots have been identified so far [70,84]. 

It should be noted that *POLD1* has been considered haploinsufficient in the literature [9,47,55]. Contrarily, a *POLD1* haplosufficiency has been recently revealed in the yeast model [94] and in germline monoallelic *POLD1* alteration carriers [95,96]. It has been speculated that a solitary heterozygous *POLD1* mutation in the exonuclease domain without concurrent mutations in other repair systems causes only a modest increase in the mutation rate [95]. In addition, Schamschula et al. suggested that *POLD1*-mutated cancers acquire the ultra-high TMB only with concurrent mismatch repair deficiency [66]. It is hypothesized that there are no cancers with an exclusive heterozygous *POLD1* mutation and only a complete loss of the two *POLD1* alleles can lead to cancer [95]. Therefore, both germline and somatic *POLD1* mutations triggering tumor formation may turn out to be biallelic [95]. Nevertheless, an attempt to organize the current knowledge and deepen understanding of POLD1 biology in carcinogenesis would be of paramount importance and could pave the way for novel anti-cancer drugs.

Whereas an overwhelming number of studies in the last decades have focused on *POLD1* pathogenic variants in a wide range of neoplasms, only a few studies have validated the prognostic and predictive utility of POLD1 mRNA and protein expression in human cancers to date. So far, *POLD1* expression and its clinical significance have been investigated in colorectal, endometrial, renal, liver, breast, lung, and thyroid cancers [13,14,15,17,19,20,21]. Nevertheless, these data are mainly limited to single-institution experiences. 

In recent years, various immunotherapy-based cancer treatments have been approved for clinical use and many additional drugs are still being developed, thus opening a new era in cancer treatment [97,98]. Moreover, ICI treatment is considered a new standard of care across many cancer indications [97]. There are several biomarkers for predicting the outcome of ICI treatment, among which expression of programmed death-ligand 1 (PD-L1), MSI status, and TMB are the most commonly used [63,97]. Moreover, high levels of TILs (especially the CD8+ T cells subpopulation [99]), common in MSI cancers, may predict the response to ICI treatment in various malignancies [100]. As some ICI recipients fail to respond to the treatment, it is therefore crucial to identify accessory molecular biomarkers for predicting the ICI response to prevent immunotherapy overtreatment [98,99,100,101,102]. 

Some reports have suggested that *POLE*/*POLD1* mutations and/or *POLE*/*POLD1* expression patterns may be useful to predict immunotherapy effectiveness [10,70]. Previous studies investigating the association of *POLE*/*POLD1* mutation with MSI status in various cancers have shown ambiguous results. Although cancers harboring *POLD1* mutation are believed to be primarily microsatellite stable, some of them may display an MSI-H phenotype [55,63,64,65,66,67,68]. Wang et al.’s study on a cohort of almost 50,000 patients with different solid cancer types assessed the impact of *POLE*/*POLD1* mutations on immunotherapy outcomes [10]. Their analysis demonstrated that among ICI-treated cancer individuals, patients with either *POLE* or *POLD1* mutations have significantly longer overall survival in comparison with those without [10]. Notably, the ICI treatment outcomes were similar, regardless of whether these mutations were located within the exonuclease domain or outside of it [10]. Regrettably, *POLE*/*POLD1* alterations cannot be independent factors for predicting the benefit from immunotherapy [101]. In addition, a study conducted by He et al. suggested that *POLE*/*POLD1* mutations in the proofreading domain may result in DNA repair defects and an exceptionally high TMB, thereby generating high neoantigen load levels that, in turn, positively correlate with the sensitivity to ICI treatment [63]. Moreover, tumors expressing higher numbers of neoantigens are associated with a better response to immunotherapy in patients with CRC, melanoma, and NSCLC [63]. However, little is known about the influence of *POLD1* expression level on the efficacy of ICI in cancer treatment. 

In the last decade, whole-genome sequencing identified specific mutational signatures that describe the patterns of mutations that arise during tumorigenesis [103,104]. Several single-base substitution (SBS) signatures have been described concerning *POLD1*-mutated tumors; SBS10c and SBS10d are linked to defective POLD1 proofreading [105], whereas SBS20 is associated with concurrent *POLD1* mutations and mismatch repair deficiency [65]. Groundbreaking studies have shown that the presence of specific SBS signatures may have prognostic and therapeutic implications [106]. Notably, Ma et al. reported that *POLD1* functional mutations generating SBS signatures associated with POLD1 activity lead to augmented immunogenicity [107]. Moreover, patients with cancers generating *POLD1*-mutation-related SBS signatures demonstrated an enhanced response to ICI treatment [107].

Given the variety of immuno-oncology treatments currently in use for therapies in patients with different cancer types, including melanoma, NSCLC, CRC, and HCC [97,108], novel research examining the relationship between the expression of the POLD1 protein and the clinical outcomes in patients receiving immunotherapy would be of great importance. Several clinical trials are ongoing to evaluate the impact of *POLD1* mutation on treatment outcomes (NCT05103969, NCT03810339, NCT03428802, NCT03491345, NCT04969029).

With the advancement of knowledge regarding cancer biology, substantial progress in the development of new diagnostic and prognostic molecular biomarkers as well as targeted therapeutic strategies against oncological diseases can be expected within the next few years. 

## Figures and Tables

**Table 1:** Changes in *POLD1* expression levels and their implications in different types of cancer.

Type of Cancer	Number of Patients	*POLD1* Status	Stage/ Grade	Detection Method	Clinicopathological Relevance	Ref.
Colorectal cancer	1069	Low	I–IV	IHC	Low protein expression had a significant association with adenocarcinoma histology, larger tumor size, and stage III tumors	[13]
Endometrial cancer	419	Low	I–IV; mainly type I EC (88.2%)	IHC	Low protein expression had a significant association with grade 1 tumors and a trend toward type I EC	[14]
Clear cell renal cell carcinoma	56	High	I–III; mainly lower grades (G1–G2 in 73.2%)	IHC	POLD1 protein expression levels in the tumor cells did not correlate with clinicopathological data Strong POLD1 protein nuclear immunoexpression in cancer cells correlated with better prognosis	[15]
Hepatocellular carcinoma	339	High	I–IV; mainly stage I and II tumors (50.1% and 24.8%, respectively)	mRNA data retrieved from the GEPIA2 database IHC	High *POLD1* expression was significantly correlated with increased levels of alpha-fetoprotein and advanced TNM stage * POLD1* upregulation was an independent indicator of poor OS DNA copy gain, low *POLD1* methylation, and downregulation of miR-139-3p may cause *POLD1* high expression *POLD1* expression correlated with the immune infiltration levels of B cells, macrophages, dendritic cells, and CD4+T cells	[17]
Breast cancer	84	High	G1–G3; invasive breast cancer treated with radical mastectomy	RT-qPCR Western blot	Increased *POLD1* gene expression was significantly associated with lymph node metastasis, histological grade, p53 status, and ki-67 index High *POLD1* gene expression was associated with poor DFS	[19]
Lung cancer	–	High	I–IV	RT-qPCR	Gradually higher *POLD1* gene expression was associated with the gradually worse prognosis of lung adenocarcinoma	[20]
Papillary thyroid cancer	286 (females in 77.7%)	Low	I–IV; mainly stage I tumors (68.3%)	IHC	Decreased POLD1 protein expression was significantly associated with the follicular variant of PTC, distant metastasis, and stage IV tumors	[21]
Lower-grade gliomas with 1p19q codeletion	280	Low	WHO grade II–III	RT-qPCR Western blot	Lower expression of the *POLD1* gene was associated with longer PFS and OS in patients who received chemotherapy	[24]
Childhood Acute lymphoblastic leukemia	90	High	–	–	In 90 matched diagnosis and relapse pairs of ALL bone marrow samples, *POLD1* expression was significantly upregulated in relapsed ALL in comparison to the newly diagnosed ALL * POLD1* upregulation was miR-520H mediated	[25]
